# Demand for Zn^2+^ in Acid-Secreting Gastric Mucosa and Its Requirement for Intracellular Ca^2+^

**DOI:** 10.1371/journal.pone.0019638

**Published:** 2011-06-15

**Authors:** JingJing Liu, Jonathan E. Kohler, Amy L. Blass, Juliet A. Moncaster, Anca Mocofanescu, Matthew A. Marcus, Eleanor A. Blakely, Kathleen A. Bjornstad, Chitra Amarasiriwardena, Noel Casey, Lee E. Goldstein, David I. Soybel

**Affiliations:** 1 Department of Surgery, Brigham and Women's Hospital, Harvard Medical School, Boston, Massachusetts, United States of America; 2 Molecular Aging & Development Laboratory, Boston University School of Medicine, Boston, Massachusetts, United States of America; 3 Advanced Light Source, Lawrence Berkeley National Laboratory, Berkeley, California, United States of America; 4 Life Sciences Division, Lawrence Berkeley National Laboratory, Berkeley, California, United States of America; 5 Channing Laboratories, Brigham and Women's Hospital, Harvard Medical School, and the Harvard School of Public Health, Boston, Massachusetts, United States of America; 6 Center for Biometals and Metallomics, Boston University, Boston, Massachusetts, United States of America; Tulane University, United States of America

## Abstract

**Background and Aims:**

Recent work has suggested that Zn^2+^ plays a critical role in regulating acidity within the secretory compartments of isolated gastric glands. Here, we investigate the content, distribution and demand for Zn^2+^ in gastric mucosa under baseline conditions and its regulation during secretory stimulation.

**Methods and Findings:**

Content and distribution of zinc were evaluated in sections of whole gastric mucosa using X-ray fluorescence microscopy. Significant stores of Zn^2+^ were identified in neural elements of the muscularis, glandular areas enriched in parietal cells, and apical regions of the surface epithelium. In *in vivo* studies, extraction of the low abundance isotope, ^70^Zn^2+^, from the circulation was demonstrated in samples of mucosal tissue 24 hours or 72 hours after infusion (250 µg/kg). In *in vitro* studies, uptake of ^70^Zn^2+^ from media was demonstrated in isolated rabbit gastric glands following exposure to concentrations as low as 10 nM. In additional studies, demand of individual gastric parietal cells for Zn^2+^ was monitored using the fluorescent zinc reporter, fluozin-3, by measuring increases in free intracellular concentrations of Zn^2+^ {[Zn^2+^]_i_} during exposure to standard extracellular concentrations of Zn^2+^ (10 µM) for standard intervals of time. Under resting conditions, demand for extracellular Zn^2+^ increased with exposure to secretagogues (forskolin, carbachol/histamine) and under conditions associated with increased intracellular Ca^2+^ {[Ca^2+^]_i_}. Uptake of Zn^2+^ was abolished following removal of extracellular Ca^2+^ or depletion of intracellular Ca^2+^ stores, suggesting that demand for extracellular Zn^2+^ increases and depends on influx of extracellular Ca^2+^.

**Conclusions:**

This study is the first to characterize the content and distribution of Zn^2+^ in an organ of the gastrointestinal tract. Our findings offer the novel interpretation, that Ca^2+^ integrates basolateral demand for Zn^2+^ with stimulation of secretion of HCl into the lumen of the gastric gland. Similar connections may be detectable in other secretory cells and tissues.

## Introduction

For many years, investigation of Zn^2+^ transport in the gastrointestinal tract has focused on nutritional requirements that maintain body stores and pathologic consequences of inadequate intake [1], [2], [3]. An overall deficiency of Zn^2+^ stores within the body has been implicated in the systemic susceptibility to infection [4], [5] and in the pathogenesis of some cancers [6], [7], [8]. Also, an important physiologic role for Zn^2+^ within the lumen of the alimentary canal has been postulated, based on the observations that supplementation of oral diets with Zn^2+^ has beneficial effects on diarrhea [9], [10] and inflammatory conditions [11], [12], [13], [14] of the gastrointestinal tract.

Recent reports have begun to explore the mechanisms that regulate cellular homeostasis of Zn^2+^ in mucosal cells of the gastrointestinal tract [15], [16], [17], [18], [19] and its potential influence on mucosal integrity and function [20], [21]. In gastric mucosa, adequate intracellular stores and luminal content of Zn^2+^ may regulate integrity of [19] and acid secretion by [18], [22] the gastric glands and enhance protection of the mucosa as a whole against acid-peptic injury [23], [24]. Little is known, however, of the content and distribution of Zn^2+^ within the mucosa, or of the mechanisms that regulate the flow of Zn^2+^ into the parietal cell during secretory stimulation.

In this study, we utilized complimentary approaches to characterize content and distribution, acquisition and demand for Zn^2+^ in gastric mucosa of the rabbit and in its individual gastric glands, under resting conditions and during secretory stimulation. Our results indicate that there is variation in content and distribution of Zn^2+^ within the gastric wall and mucosa. We find that, *in vivo* and *in vitro*, the mucosa and individual gastric glands are capable of extracting Zn^2+^ from extracellular sources even when its concentration may be in the nanomolar range. In the isolated gastric gland, a multicellular model of epithelial secretion, we find that basolateral uptake of Zn^2+^ is modulated by [Ca^2+^]_i_ during stimulation with agonists of apical secretion of acid. Our findings thus suggest a novel role for classical second messenger pathways in integrating secretory functions of the apical membrane with supply functions across the basolateral membrane, and may be applicable to a variety of epithelial systems.

## Methods

### Animals and tissue procurement

Anesthesia and euthanasia for New Zealand White rabbits were approved according to policies of Harvard Medical School (Harvard Medical Area (HMA) Standing Committee on Animals; Protocol 03359). As described previously [15], [18], rabbits (female, ∼2 kg) were anesthetized with ketamine and pentobarbital, undergoing midline laparotomy in order to harvest stomach tissues and glands. For studies of fixed tissue, full thickness squares (7 mm to 10 mm each side) of gastric wall from the acid secreting regions (body/fundus) were obtained, then cut into small strips and frozen in liquid nitrogen. For gland isolations, the aorta was perfused retrograde with warmed (37°C) phosphate-buffered saline, as described previously [15]. The gastric mucosa was separated from underlying muscularis. Isolated glands were prepared using published methods [15], [25]. Collagenase Type I (Sigma Chemical, St. Louis, MO) was used for ∼60 min digestion with BSA in Dulbecco's Modified Eagle Medium (DMEM, Sigma Chemical, with 100 µM cimetidine, pH 7.4). Glands were used within 8 hr of isolation.

#### Micro X-Ray Fluorescence Studies

To survey the distribution of metals in the gastric mucosa, we utilized micro X-Ray Fluorescence microscopy (μXRF) [26]. In this technique, a micron-size X-ray beam is rastered over the sample. The incident X-rays excite fluoresacence from elements in the sample such as Zn provided the incident energy is above the relevant absorption edge. For Zn, the edge is at 9.66 keV and the Ka fluorescence at 8.62 keV. A solid-state energy-resolving detector picks up the fluorescence and the counts in energy regions corresponding to elements of interest are recorded for each pixel. Our samples are thin enough to be transparent to both the incident and fluorescence X-rays from the detected elements. In this regime, the signals are proportional to the column densities (µg.cm^2^) of each element, with a different sensitivity for each element.

**Figure 1:**
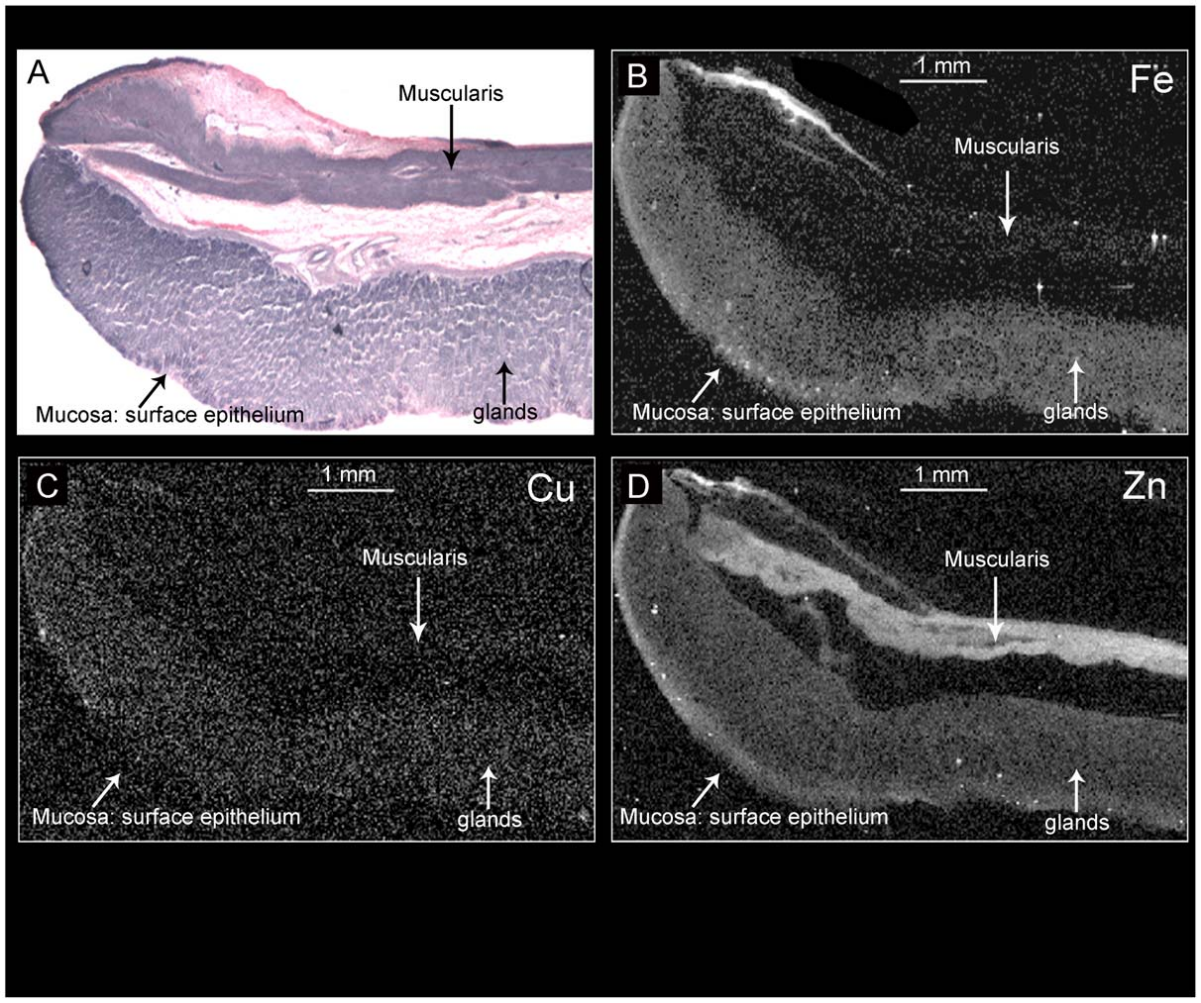
Mapping metal divalent cations in rabbit gastric mucosa. Figure 1A visualizes the section of interest with hematoxylin/eosin staining. Figure 2B, 2C and 2D provide maps at low resolution (20×20 µm^2^ pixel) of Fe, Cu and Zn. In these images, white and black indicate high and low levels of the metal, respectively.

**Figure 2:**
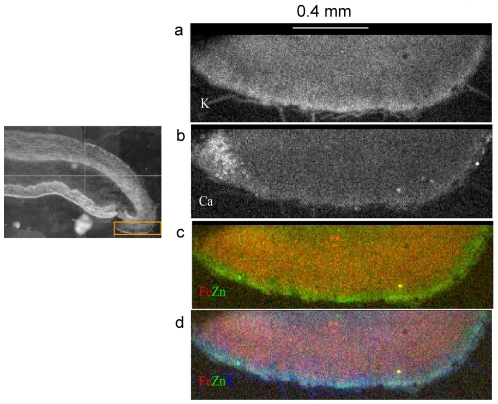
Comparisons of the distributions of Zn^2+^ and other abundant cation species in a smaller region of gastric mucosa. Region of interest is shown in the inset (Figure 2A). The pixel size is 5×5 µm^2^. See text for interpretation of the color images.

For full thickness samples, a novel fast-freeze method was developed for near instantaneous tissue vitrification. Fixation and mounting of oriented specimens was performed on silicon wafers (Platypus Technologies, Madison, WI). Silicon substrates were used because they exhibit low background signals in μXRF and have high thermal conductivity. Sequential cryosections (50 µm) were stained with hematoxylin/eosin and then analyzed by X-ray fluorescence microscopy on Beamline 10.3.2, Advanced Light Source, Lawrence Berkeley National Laboratory, Berkeley, CA. [27]. Samples were transported to the Beamline on dry ice and mounted on a Peltier stage at −27°C without being permitted to thaw. The conditions for the map shown in Figure 1 b–d were: incident energy 11.5 keV, dwell time 100 msec/pixel, pixel size 20×20 µm^2^, and incident beam size 16×7 µm^2^ FWHM. For the finer map in Figure 2, the parameters were: incident energy 12.5 keV, dwell time 400 msec/pixel, pixel size 5×5 µm^2^. and incident beam size 7×7 µm^2^ FWHM. Fluorescence counts were recorded in energy bands corresponding to K, Ca, Fe, Cu, Ni and Zn, though no significant Ni was found. Since the signals from some elements appear in more than one band, e.g. the Kβ fluorescence of Cu in the Zn Kα band, certain fractions of the signal from such interfering channels were subtracted from the channels interfered with. The coefficients for this subtraction were derived by mapping standards containing some but not all relevant elements, e.g. Cu with no Zn.

#### High Resolution Magnetic Sector Inductively Coupled Plasma Mass Spectrometry (HR-ICP-MS)

Among the five stable zinc isotopes, ^64^Zn, ^66^Zn, ^66^Zn, ^67^Zn and ^70^Zn. ^70^Zn is the most commonly isotopic tracer due to its low natural abundance and was used for all described experiments. The natural relative abundance for ^70^Zn and ^68^Zn are 0.6% and 18.8% respectively with an isotopic ratio of 0.0319 and any increase in this isotopic ratio is indicative of ^70^Zn uptake in biospecimens. Unless otherwise noted, all reagent and stock solutions were prepared in ultra-trace metal grade nitric acid (Fisher Brand, Fisher Scientific, USA) made from de-ionized water (Millipore, Billerica, MA, USA) in order to match the acid matrix resulting from the sample preparation procedure. Zinc enriched in ^70^Zn (99%) was obtained (Cambridge Isotope, MA, USA) and was dissolved in HCl and diluted with de-ionized water to prepare an ^70^Zn-enriched spiking solution of 1000 mg/L in 1% HCl/citrate. This stock solution was used to prepare standard and sample solutions for all experiments. Standard-sized tissues samples (∼25 mm^3^) or volumes of isolated glands (0.3 ml or ∼2,000 glands, following settling and aspiration of supernatant media) were acid digested in aliquots of concentrated nitric acid and made up to a total volume 2 ml using de-ionized water. Analysis for isotopes ^68^Zn and ^70^Zn were performed concurrently on 1 ml samples, at the ICP-MS facility of the Institute of Marine and Coastal Sciences, Rutgers the State University, New Brunswick, NJ. An analytical methodology was developed to scan zinc isotopes using a double-focusing, single-collector Thermo Element 2 ICP-MS (Thermo, Waltham, MA, USA). Settings are shown in Table 1. Sample solutions were introduced into a spray chamber by a low-flow nebulizer using argon as a carrier gas. The signal intensity was obtained by integration of the counting signal of the scanning mass over a 2–4 min acquisition period. Sample blanks were taken into account and a natural abundance zinc standard solution was analyzed in between sample runs for quality control purposes [28]. Thus, in each sample the ratio for ^70^Zn/^68^Zn was calculated as an index of enrichment [28].

**Table 1:** Typical Element 2 ICP-MS Instrumental parameters for the determination of zinc isotope ratios.

**Forward rf power**	**1050 W**
**Reflected rf power**	**<2 W**
**Coolant gas flow rate**	**16 L/min**
**Auxiliary gas flow rate**	**1 L/min**
**Nebulizer gas flow rate**	**0.8–1.2 L/min**
**Focus lens**	**−700–−900 V**
**Y-Deflection lens**	**−1–−10 V**
**Mass range**	**^68^Zn, ^70^Zn**
**Dwell time/point**	**0.005 s**
**Points/peak**	**10**
**Scans/measurement**	**1500**
**Solution uptake rate**	**20–100 µL/min**
**Scan mode**	**E-scanning, peak-hopping**

#### Fluorescence Microscopy or Plate-Reader Fluorometry for intracellular [Zn^2+^] using fluozin-3

Concentrated preparations of freshly isolated gastric glands were diluted in DMEM to a final concentration 1.875% and loaded for 30 min to 40 min with fluozin-3AM (8 µM) [18], [19]. Following transfer to glass coverslips and equilibration in standard Ringer's solutions, fluorescence imaging of individual glands was performed as described previously [18]. Glands loaded with fluozin-3AM were excited at 488 nm with emission measurement at 520 nm. Fluorescence was monitored concurrently in 4 to 8 individual parietal cells in each isolated gland. Digital images of glands were captured using a CCD camera (Hamamatsu ORCA-ER). In order to take into account variations in starting levels between individual cells, responses were reported as a normalization to starting values (F/F_0_) [18], [19].

For high-throughput fluorometry, aliquots of glands (200 µl, ∼2000 glands/well) were transferred to 96 well plates (total volume 0.50 ml) following loading of dye in media and experimental manipulations in standard (in mM: 145 NaCl, 2.5 KH_2_PO_4_, 1.0 MgSO_4_ or 1.0 MgCl_2_, 1.0 CaCl_2_, 10 HEPES, and 10 glucose, pH 7.4; concentration of Zn^2+^ measured by atomic absorption is 1.2 µM, kindly determined by Dr. Shannon Kelleher, Pennsylvania State University, University Park, PA) or modified Ringer's solutions. Readout of fluozin-3 fluorescence was performed (Excitation 485 nm/Emission 520 nm) after correction for background fluorescence of solutions and unlabelled glands [29], [30]. Fluorometry was performed using a Synergy™ 2 Multi-Mode Microplate Reader (BioTek Instruments, Inc.). When comparisons to the starting fluorescence levels were required, responses were reported as a normalization to starting values (F/F_0_) [18], [19]. When comparing responses between groups at a single time point, the responses in total fluorescence are reported.

### Special Reagents

Thapsigargin and TPEN {tetrakis-(2-pyridylmethyl)ethylenediamine} were obtained from Molecular Probes (Eugene, OR) and were dissolved in Ringer's solutions directly. Nigericin, DTPA (diethylenetriaminepentaacetic acid) and pyrithione were obtained from Sigma-Aldrich and was dissolved under alkaline conditions, then added to Ringer's solutions for final dilutions of 1∶1000. All solutions were checked for changes in pH and adjusted if necessary to baseline pH. For Ringer's, calculations of free and bound concentrations of Ca^2+^, Zn^2+^, TPEN, and EGTA were performed using the internet-based WEBMAXSTANDARD program (http://www.stanford.edu/~cpatton/webmaxc/webmaxcS.htm).

### Data Summary and Statistical Analysis

In the microscopy imaging system, fluorescence intensities were monitored continuously throughout each experiment (SimplePCI software). At discrete time intervals, measurements were summarized as means ± SE. For comparison between treatments, unless stated otherwise, measurements in individual parietal cells (4 to 8 cells identified in each isolated gland) were combined to provide a single integrated value at each time point for each gland. Unless stated otherwise, comparisons were performed using analysis of variance for sequential or multiple measurements (Kruskal-Wallis and Tukey test for pairwise multiple comparisons), using purchased software (Sigma Stat, Version 3.5).

## Results

### Metal Maps of Gastric Mucosa using μXRF

Shown in **Figure 1** are metal maps for individual divalent metals (Zn, Cu, Fe), demonstrating relative differences in content and distribution in the mucosa. **Panel 1A** provides a standard tissue frozen section, stained with hematoxylin and eosin, corresponding to a neighboring section that was used to generate metal maps (**Panel 1B:** Fe; **Panel 1C:** Cu; **Panel 1D:** Zn). In these maps, white represents the maximum intensity while black areas show little or none of the relevant element. Of note, consistently strong signals for the Fe and Zn are detected in region of the surface epithelium, near the luminal interface; signals for Cu are very minimal throughout the section although perhaps more concentrated in the surface epithelium. Relatively homogeneous signals for Fe are observed throughout the glandular mucosa, deep to the surface epithelium, in areas known to be populated by mitochondria-rich parietal cells [31], [32]. Signal for Zn appears to be distributed within the mucosa much as that for Fe. In contrast, dense content of Zn was detected in the region of the myenteric nerve plexus, lying between the layers of muscle, whereas there was relatively little signal for Cu or Fe. As a control, samples were interrogated for Ni, a metal component of the microtome knife. No Ni was detected (data not shown) indicating that contamination of the specimen with extraneous metals was unlikely.

To further define distribution of Zn within the glandular region of the mucosa (**Figure 2**), a region 1.8 mm in longest dimension was scanned in more detail, comparing the distribution of Zn with other physiologically relevant elements (K, Ca, Fe). Data for K and Ca are presented in grayscale as in Figure 1, and data for Zn, Fe and K are presented in various combinations as bicolor or tricolor maps. In the bottom-most map (Figure 2d) the red intensity of each pixel represents the relative amount of Fe, the green the amount of Zn and the blue the amount of K. Thus, blue-green colors represent areas containing relatively large amounts of K and Zn together. The surface epithelium is notable for a relatively high content of Zn, compared to the glandular regions deep in the mucosa. Content and distribution of Zn^2+^ is homogeneous throughout the glandular region.

### Measurements of ^70^Zn^2+^ uptake in gastric mucosa and isolated gastric glands

**Figure 3:**
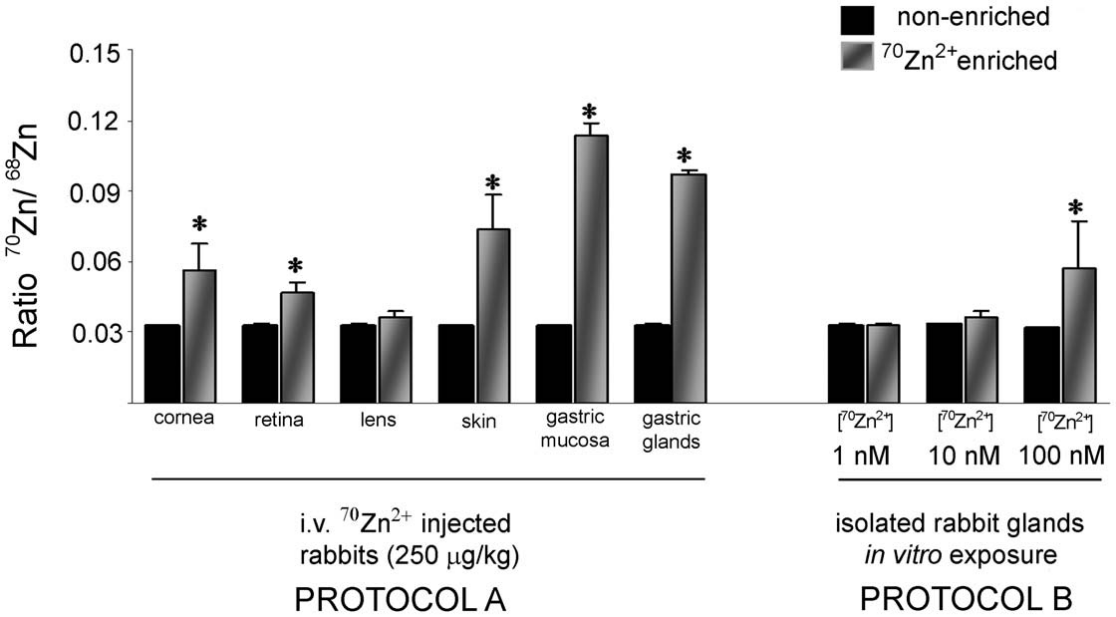
Acquisition of ^70^Zn in tissues and isolated gastric glands of the rabbit. Measurements of isotopic Zn species by ICP-MS are reported as the ratio of ^70^Zn/^68^Zn (no dimensions) means ± SD, *p<0.05 compared to control measurements. **Protocol A**. Measurements in tissues harvested from rabbits: three control (black columns) and in three rabbits 18 hrs after intravenous infusion of ^70^ZnCl_2_ (250 µg/kg). **Protocol B**. Measurements performed in aliquots of gastric glands exposed to standard Ringer's containing EGTA, Ca^2+^ (calculated free concentration 1 mM) and reagent ZnCl_2_ (control) or ^70^ZnCl_2_ (calculated free concentrations 1 nM, 10 nM or 100 nM).

To demonstrate that gastric mucosa acquires Zn^2+^ from the circulation, *in vivo*, three pairs of rabbits were injected intravenously with ^70^Zn^2+^ in sterile saline (ZnSO_4_, 250 µg/kg) to one animal and saline alone to the other. Gastric mucosa was harvested 24 hr later, some preserved for analysis and the rest processed for isolation of individual gastric glands. The relative abundance in nature for ^70^Zn and ^68^Zn are 0.6% and 18.8%; and the ratio of ^70^Zn/^68^Zn is 0.0319. Any enrichment of ^70^Zn reflected in a higher ratio would reflect uptake in tissues and individual glands. [33]. As shown in **Figure 3**, ICP-MS analysis of whole mucosa and isolated glands revealed appropriate ratios of ^70^Zn∶^68^Zn in saline injected animals, along with significant uptake of ^70^Zn^2+^ in whole gastric mucosa and isolated gastric glands. Avidity of gastric mucosa for ^70^Zn^2+^ is higher than that of peripheral tissues such as skin, retina, cornea and much higher than the avascular lens.

In *in vitro* studies, we evaluated potential threshold levels of extracellular [Zn^2+^] which would permit detection of ^70^Zn^2+^ uptake by gastric mucosa. Isolated gastric glands were exposed to increasing concentrations of ^70^Zn^2+^ in Ringer's solutions (containing EGTA 0.3 mM, with Ca^2+^ adjusted to keep its free concentration 1 mM), with total content of added Zn^2+^ calculated to provide a free [Zn^2+^] of 1 nm, 10 nM or 100 nM. After 1 hour, glands were processed for ICP-MS. In unstimulated glands, evidence of accumulation started to become evident during exposure to extracellular [Zn^2+^] at 10 nM (**Figure 3**), although accelerated and significant uptake was more consistently observed when [Zn^2+^] reached 100 nM. These data indicate that basolateral uptake processes operate at low threshold concentrations for free extracellular Zn^2+^, consistent with those that have been predicted [34] and measured [35], [36] in the circulating plasma.

### Fluorescence microscopy in individual parietal cells of isolated gastric glands: dependence of Zn^2+^ uptake on [Ca^2+^]_i_

**Figure 4:**
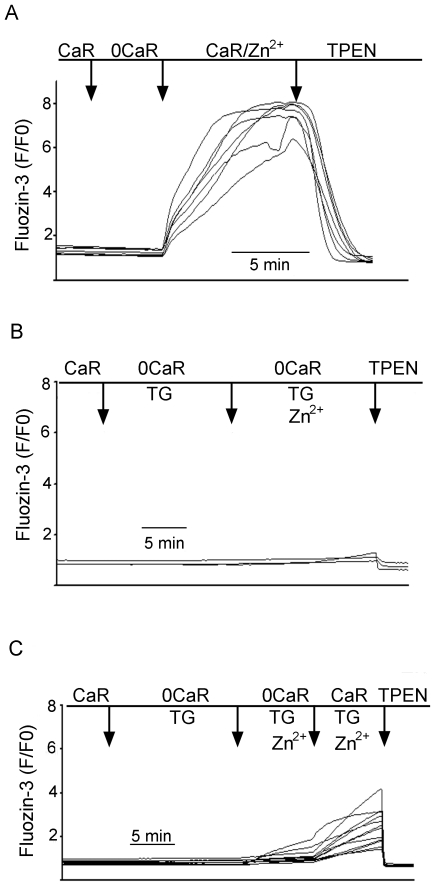
Recordings from imaging studies in individual isolated gastric glands of [Zn2+]I during deprivation and restoration of Ca^2+^. **Panel 4A:** A single gland with 7 parietal cells imaged, exposed first to Ringer's solution {Ca^2+^ 1 mM, no added Zn^2+^}, then Ringer's solutions depleted of Ca^2+^ but containing Zn^2+^ 10 µM, then Ringer's solutions containing both 1 mM Ca^2+^ and 10 µM Zn^2+^. Recording is terminated with addition of TPEN to confirm that signals are due to Zn^2+^. Note variable responses in individual cells. In 15 of 17 studies (88%), loading of Zn^2+^ was observed in the presence of Ca^2+^. Among the 15 glands responding, the mean increase over baseline was 157%+3% (P<0.0001). **Panel 4B:** A single gland cell (3 cells), exposed initially to thapsigargin (2 µM) and Ca^2+^-depleted Ringer's solution, then to Ringer's containing thapsigargin and 10 µM Zn^2+^ in the absence of Ca^2+^. In 49 glands (3–8 cell per gland), no loading response to 10 µM Zn^2+^ was observed in the absence of extracellular Ca^2+^, more than 3% above baseline. **PANEL 4C**. A single gland (12 cells) exposed to initial conditions similar to those in Panel 2B, but with 1 mM Ca^2+^ present. Among 24 glands, the mean response was 60%+1% over baseline. In each recording, the experiment is terminated with addition of TPEN to confirm that signals are due to Zn^2+^. These findings indicate that extracellular Ca^2+^ partially restores Zn^2+^ loading and that uptake of Zn^2+^ across the basolateral membrane of the parietal cell is regulated by [Ca^2+^]_i_.

Flux measurements of ^70^Zn^2+^ reliably measure uptake in relation to small extracellular concentrations; however, they do not allow continuous monitoring of intracellular Zn^2+^ accumulation. To explore the conditions regulating acquisition of Zn^2+^ in real time, glands were loaded with fluozin-3 (Ex 485 nm/Em 520 nm) in order to monitor changes in intracellular concentration of Zn^2+^ {[Zn^2+^]_i_} in individual parietal cells [18]. Following transfer to coverslips on a microscope stage, fluorescence was monitored during exposure to standard Ringer's solutions containing free [Zn^2+^] of 10 µM to 50 µM. As shown in **Figure 4A**, exposure to standard Ringer's containing an additional 10 µM Zn^2+^ leads immediately to increases in [Zn^2+^]_i_, reaching plateaus within 10 minutes. These responses were abolished during exposure to low concentrations of a membrane-permeable chelator, TPEN (10 µM), indicating that Zn^2+^ is the source of the fluorescence signal within the cell [37].

Utilizing fluozin-3 measurements, previous studies in our laboratory [18] have consistently detected *intracellular* accumulation of labile Zn^2+^ in isolated gastric glands when *extracellular* Zn^2+^ was at least 50 µM, and this was observed in the presence or absence of extracellular Ca^2+^. We also observed an increase in the rate of Zn^2+^ uptake when glands were exposed to the secretagogue, forskolin (10 µM), a finding consistent with the idea that stimulation of acid secretion across the apical membrane leads to increased demand for Zn^2+^ across the basolateral membrane. These observations were corroborated by preliminary studies indicating that exposure to forskolin (10 µM) enhances influx of isotopic ^70^Zn^2+^, more than three-fold over baseline influx (extracellular concentration 50 µM, data not shown). In these *in vitro* studies, when extracellular Ca^2+^ was removed, responses became less consistent, particularly if extracellular Zn^2+^ concentrations were 10 µM or lower. These observations led us to hypothesize that uptake of Zn^2+^ into the parietal cell might be connected to intracellular Ca^2+^ homeostasis.

To assess whether intracellular uptake of Zn^2+^ requires the presence of Ca^2+^, a protocol was devised to totally deplete Ca^2+^ from extracellular solutions and intracellular stores. Glands pre-loaded with fluozin-3 were perfused with Ca^2+^-depleted Ringer's (0.3 mM EGTA) containing 2 µM thapsigargin to deplete the intracellular Ca^2+^ store, followed by exposure to 10 µM Zn in Ca^2+^-depleted Ringer's. As shown in a representative recording from 5 parietal cells in a single gland in **Figure 4B**, Zn^2+^ accumulation was abrogated under these conditions, indicating a requirement for intracellular Ca^2+^. Exposure to thapsigargin depletes intracellular, membrane-bound stores of Ca^2+^, but activates store-operated channels at the plasma membrane [38], [39]; and exposure to both thapsigargin and physiologic concentrations of extracellular Ca^2+^ lead to increases in [Ca^2+^]_i_ without any re-filling of stores [40]. We therefore asked whether uptake of extracellular Zn^2+^ can occur when intracellular, membrane–bound stores are depleted but store-operated entry of Ca^2+^ is present (**Figure 4C**). Gastric glands loaded with fluozin-3 were exposed first to Ringer's containing thapsigargin (2 µM) and no added Ca^2+^ to deplete intracellular and extracellular sources of Ca^2+^. Under these conditions no accumulation was observed in the presence of extracellular Zn^2+^ (10 µM). When Ca^2+^ was subsequently restored to the perfusate (1 mM) in the presence of thapsigargin, increases in [Zn^2+^]_i_ were observed (**Figure 4C**). Thus, the cell's ability to take up extracellular Zn^2+^ requires some level of intracellular Ca^2+^ content, but not necessarily the presence of functionally intact intracellular stores of Ca^2+^.

### Ca^2+^-dependent accumulation of intracellular [Zn^2+^] is due to influx of Zn^2+^ from extracellular sources

**Figure 5:**
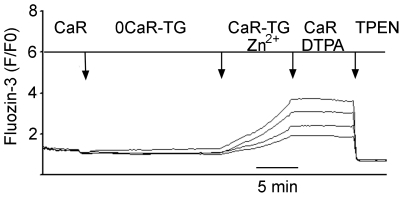
Ca^2+^-dependent increases in [Zn^2+^]_i_ are attributable to influx from extracellular sources. Recording from imaging of a single gland (4 individual parietal cells) loaded with fluozin-3. The recording begins with perfusion in standard Ca-Ringers solution, followed by 0 Ca Ringer's containing 2 µM thapsigargin, which depletes intracellular calcium stores. During exposure to 10 µM Zn^2+^, in the presence of Ca^2+^, a monotonic increase in [Zn^2+^]_i_ follows, similar to that observed in **Figure 4C**. With addition of an excess of DTPA (100 µM), a high affinity and membrane impermeable chelator of heavy metals (but not Ca^2+^ or Mg^2+^), the accumulation of Zn^2+^ is arrested. In 18 cells monitored in 5 glands, this arrest was always observed, demonstrating that Ca^2+^ dependent loading of Zn^2+^ was due solely to influx from extracellular sources.

We next performed studies to confirm that Ca^2+^-dependent increases in intracellular Zn^2+^ is due to influx from extracellular sources rather than release from intracellular pools. Glands loaded with fluozin-3 were exposed initially to Ringer's solutions containing and thapsigargin (2 µM) and no added Ca^2+,^ in order to deplete intracellular stores. These solutions also contained DTPA (10 µM), a chelator of Zn^2+^ that is membrane-impermeable and therefore depletes free Zn^2+^ in the extracellular perfusate but not within the cell. Under these conditions, no increase in [Zn^2+^] was observed. Glands were then exposed to solutions containing Zn^2+^ (10 µM) and Ca^2+^ (1 mM) and, as expected, increases in intracellular Zn^2+^ were observed. These increases were, however, arrested when the chelator DTPA (10 µM) was added (**Figure 5**). These findings indicate that the increases in [Zn^2+^]_i_ are due to influx from extracellular sources and not release from intracellular pools.

### Fluorometric measurements of [Zn^2+^]_i_: evidence for Ca^2+^/Zn^2+^ exchange

In fluorescence microscopy, there is a possibility of inadvertent bias in selection of individual glands for study. To be confident of the overall characteristics of the response to depletion and restoration of [Ca^2+^]_i_, we adapted our fluorescence-based protocol of monitoring demand for Zn^2+^, using a 96-well platform, which permits assessment of responses in populations of glands (∼2,000 glands/well).

**Figure 6:**
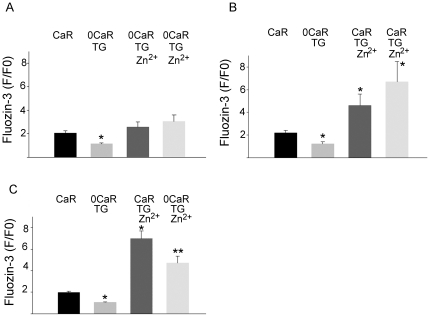
Influence of Ca^2+^ availability on uptake of Zn^2+^ by populations of isolated gastric glands. Studies were performed to determine whether plate-reader-based measurements were capable of detecting Ca^2+^-dependent changes in [Zn^2+^]_i_. Rabbit gastric glands were pre-loaded with fluozin-3AM for 45 mins. Aliquots of glands (N = 6 each group) were transferred to 96 well plates (∼2,000/well) coated with polylysine to promote adherence. Solutions were exchanged with fresh solutions at the intervals noted below to assess response of Zn^2+^ loading when intracellular Ca^2+^ stores had been depleted. Fluorescence intensity was measured at the end of each treatment and compared to baseline measurements under control conditions (F/F_0_ where baseline is set at arbitrary ratio 2.0). Measurements reported as means ± SEM. (* p<0.001 ** p<0.05 compared to baseline Ringer's measurements in each set). **Panel 6A:** Measurements of [Zn^2+^]_i_ in gastric gland preparations exposed, in sequence, to Ca-Ringer's (15 mins); 0Ca-Ringer's-thapsigargin (35 mins); 0Ca-Ringer's-10 µM Zn (30 mins); 0Ca-Ringer's-10 µM Zn (an additional 30 mins). **Panel 6B:** Measurements of [Zn^2+^]_i_ in gastric gland preparations exposed, in sequence, to Ca-Ringer's (15 mins); 0Ca-Ringer's (35 mins)-thapsigargin; Ca-Ringer's-10 µM Zn (30 mins); Ca-Ringer's 10 µM Zn (an additional 30 mins). **Panel 6C:** Measurements of [Zn^2+^]_i_ in gastric gland preparations exposed, in sequence, to CaR (15 mins); 0 Ca-Ringer's-thapsigargin (35 mins); Ca-Ringer's 10 µM Zn (30 mins); 0 Ca-Ringer's-10 µM Zn (30 mins).

A first set of studies (**Figure 6A**) was performed to confirm that accumulation of Zn^2+^ in a population of glands was impaired in the absence of Ca^2+^. Glands were exposed, in sequence to: standard Ringer's solutions containing 1 mM Ca^2+^ (CaR, 30 min), then Ringer's depleted of Ca^2+^ and containing 300 µM EGTA (0CaR TG, 30 min), then 0CaR TG solutions containing 10 µM Zn^2+^ (0CaR TG 10 uM Zn) for 30 min with replacement by fresh solution at 30 min. As shown, resting levels of [Zn^2+^]_i_ were decreased and very little uptake was detected when extracellular and intracellular sources of Ca^2+^ had been depleted.

A second set of studies (**Figure 6B**) was then performed to confirm that uptake of Zn^2+^ could be observed despite functional impairment of intracellular Ca^2+^ stores. To establish baseline conditions with depletion of intracellular stores, glands were exposed to CaR (30 min) and then 0CaR with TG (30 min). Glands were then exposed to solutions in which Ca^2+^ was restored (CaR, TG and Zn^2+^ 10 µM) for 30 min, with replacement by fresh solution at 30 min. As in studies of individual glands (**Figures 4** and **5**), when Ca^2+^ was restored to the system, uptake of Zn^2+^ was significantly enhanced and continued to the last period of observation (**Figure 6B**).

A third set of studies (**Figure 6C**) was then performed to confirm reversibility of Ca^2+^-dependent uptake of Zn^2+^. Baseline conditions were established with depletion of intracellular stores (CaR for 30 min followed by 0CaR-TG for 30 min). Glands were then exposed to solutions in which Ca^2+^ had been restored (CaR, TG and Zn^2+^10 µM) for 30 min, followed by exposure to solutions in which Ca^2+^ was again depleted (0CaR, TG and Zn^2+^ (10 µM) for 30 min. As previously observed, when Ca^2+^ was restored to the system, uptake of Zn^2+^ was significantly enhanced; however, when Ca^2+^ was again removed accumulation of Zn^2+^ was impaired and actually decreased. These findings suggest that Zn^2+^ accumulation is responsive to Ca^2+^ availability in the cytoplasm, by a mechanism that is consistent with exchange of intracellular Ca^2+^ for extracellular Zn^2+^.

### Demand for extracellular Zn^2+^ during secretory stimulation

In a final series of studies, we evaluated demand for extracellular Zn^2+^ during maximal secretory stimulation, exposing glands to the muscarinic agonist carbachol (CCh, 1 mM) and the cAMP/PKA agonist forskolin (FSK, 10 µM) [25], [41], [42]. Glands were loaded with fluozin-3, then pre-stimulated with CCh/FSK or vehicle (1∶1000 DMSO) in media for 30 min before being loaded into wells containing Ringer's solutions with 10 µM Zn^2+^. Responses were read 10 minutes after placement in these solutions, in order to obtain immediate estimates of labile Zn^2+^ accumulation within the cell.

**Figure 7:**
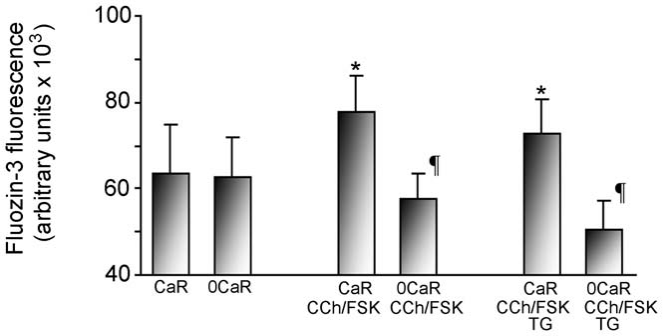
Rapid throughput assay showing influence of Ca^2+^availability on uptake of Zn^2+^in populations of isolated gastric glands, during exposure to secretagogues. Aliquots of fluozin-3 loaded glands (n = 8 each group) were incubated in wells for 45 min in Ca-Ringer's or 0Ca-Ringer's solutions under control conditions, during exposure to secretagogue combination (carbachol CCh 100 µM, forskolin FSK, 10 µM), or during exposure to secretagogue combination and thapsigargin TG, 10 µM. Glands were then exposed for 10 min to solutions containing Zn^2+^ 10 µM to monitor content. Results reported in total fluorescence units, means ±SEM, *p<0.05 compared to Ringer control, ¶p<0.05 compared to CaR conditions. ***Designations:*** a) Ca-Ringer's 45 min then Ringer's/Zn^2+^10 µM 15 min (**CaR**); b) 0Ca-Ringers 45 min/Zn^2+^10 µM 15 min (**0CaR**); c) Ca-Ringer's, carbachol 100 µM, forskolin 10 µM for 45 min then Ca-Ringer's, carbachol 100 µM, forskolin 10 µM, Zn^2+^10 µM 15 min (**CaR-CCh-FSK**); d) 0Ca-Ringer's/2 µM Thapsigargin 45 min then 0Ca-Ringer's/Thapsigargin 2 µM/Zn^2+^10 µM 15 min (**0CaR-CCh-FSK**); e) Ca-Ringer's, carbachol 100 µM, forskolin 10 µM, 2 µM, thapsigargin for 45 min then Ca-Ringer's, carbachol 100 µM, forskolin 10 µM, thapsigargin 2 µM/Zn^2+^10 µM 15 min (**CaR-CCh-FSK/TG**); f) 0Ca-Ringer's/2 µM Thapsigargin 45 min then 0Ca-Ringer's/Thapsigargin 2 µM/Zn^2+^10 µM 15 min (**0CaR-CCh-FSK/TG**).

As shown in **Figure 7**, under un-stimulated conditions, intracellular content of Zn^2+^ was not influenced by the absence of extracellular Ca^2+^. During exposure to secretory agonists (CCh/FSK), accumulation of Zn^2+^ was significantly enhanced if Ca^2+^ was present in the Ringer's solution, but significantly impaired if Ca^2+^ was absent. These findings confirm that, during secretory stimulation, intracellular content of Zn^2+^ depends on influx of extracellular Ca^2+^. In an additional experiment, also shown in **Figure 7**, we found that pretreatment with thapsigargin (TG) did not impair accumulation of Zn^2+^ during exposure to CCh/FSK, if Ca^2+^ was present in the Ringer's solution, indicating that functional intracellular stores of Ca^2+^ are not, *per se*, required for the ability of the gland to take up Zn^2+^ during secretory stimulation. In contrast, pretreatment with TG in Ca^2+^-free Ringer's enhanced the failure of the glands to preserve content of Zn^2+^, a finding consistent with the hypothesis that accumulation of Zn^2+^ is even more completely impaired by profound depletion of Ca^2+^ from the extracellular compartment, intracellular stores and the cytoplasm.

## Discussion

Work reported previously from our laboratory indicates that the transport of Zn^2+^ by the parietal cell is a critical feature of its ability to regulate acidity within its secretory compartment, which includes the tubulovesicles of the parietal cell and the lumen of the gland. Chelation of Zn^2+^ within these compartments led to marked alkalization, suggesting that the presence of Zn^2+^ insulates these compartments against leakage of H^+^ ions [15]. Recently we have reported evidence that the secretory compartments serve as a reservoir for Zn^2+^, accumulating it in response to proton gradients and failing to accumulate it when secretion is blocked by proton pump inhibition [18].

The studies reported here provide three sets of observations with respect to utilization of zinc within the gastric mucosa: first, as demonstrated by μXRF, that zinc accumulates differently in distinct micro-anatomic regions of the mucosa; second, as observed following *in vivo* intravenous injection, the non-radioactive isotope ^70^Zn^2+^ accumulates readily within both gastric mucosa and in the acid-secreting gastric glands; and third, as suggested by *in vitro* studies of isolated gastric glands, that exposure to well-recognized secretory agonists accelerates the uptake of Zn^2+^ from the nutrient side of the epithelium and requires intact intracellular stores of Ca^2+^. The studies reported here refine and extend the concept that demand for Zn^2+^ from the circulation and nutrient compartment may be regulated acutely by alterations in H^+^ secretion to the lumen.

### Heterogeneous distribution of Zn^2+^ within the gastric mucosa

To our knowledge, this is the first report of the use of μXRF to evaluate the distribution of Zn^2+^ and other cations in the mucosa of a specific region of the gastrointestinal tract. The basis of the method is fluorescence produced by synchrotron-derived x-rays, which can be focused to localize elemental content within isolated cells [43], [44] or tissues [45], [46]. *In vitro*, quantification within individual cells and resolution of individual isotopes of different metals is feasible [26], [43], [44]. However, interpretation of differences in distribution within tissues depends on unique structural features [45] or co-localizing markers [46] for cellular or sub-cellular areas of interest.

The gastric mucosa is divided into two spatially and functionally distinct regions [31], [47], [48]: the gastric glands, which secrete acid and pepsinogen; and the surface epithelium, which secretes mucus and bicarbonate and is thought to provide the protective barrier against back-diffusion of luminal H^+^ ions. Within the gastric gland, the region toward the surface is populated with mucus neck cells; the middle region is dominated by the acid-secreting parietal cells, and the deepest regions harbor pepsinogen-secreting chief cells [47]. The precise localization and quantification of metal signals in the stomach awaits development of co-localizing markers suitable for use in tissues prepared for μXRF. However, the distinct histologic structure of the mucosa permits general conclusions regarding distribution of zinc in the regions of the gastric glands and the surface epithelium. Our μXRF studies thus demonstrate a consistent signal for zinc within the glandular regions. At the same time they do not suggest major variation within different regions of the gastric gland.

Curiously, we find higher levels of Zn^2+^ present at the interface of the lumen and the surface epithelium (**Figures 1** and **2**), more consistently than other metals such as Cu^2+^ and Fe^2+^, and not in conjunction with other intracellular cations (K^+^, Ca^2+^) that are more likely to be free and labile than bound. This observation offers the possibility that Zn^2+^ is secreted with the mucus by the surface epithelium or, possibly, is selectively trapped within the mucus layer after discharge into the gastric lumen with secretion. It is tempting to speculate that associations of Zn^2+^ or other metals with the mucin layer might be important in its structure or ability to resist back-diffusion of H^+^ ions. The development of a method for evaluating distribution of metal ion species in a complex and highly hydrated mucosal surface provides opportunities to evaluate alterations in content and distribution of metal ion species under pathologically relevant conditions, for example, in response to systemic stress or during infestation with pathogenic organisms such as *Helicobacter pylori*.

### Demonstrating and monitoring the demand for Zn^2+^: *in vivo* and *in vitro* studies

The concept of “demand” for any nutrient is based on the need of a cell, tissue or organism to maintain homeostasis. In studies utilizing sector field ICP-MS, following injection of a non-radioactive and naturally scarce isotope, ^70^Zn^2+^, uptake is demonstrated within the mucosa as a whole and in individual glands. Such movements can be tracked within hours of injection into the circulation and provide evidence that demands of the tissues are readily replenished by movement of Zn^2+^ from the circulation into the mucosa.

To more precisely characterize the avidity of individual glands, we developed an *in vitro* assay to monitor uptake of ^70^Zn^2+^ by isolated glands, observing that it can be detected within minutes when extracellular concentrations are in the 10 nanomolar range and very consistently when concentrations are 100 nM. Moreover, rates of uptake are accelerated during stimulation with a powerful secretory agonist, forskolin. The use of rare, non-radioactive isotopes such as ^70^Zn^2+^ permits investigation of Zn^2+^ distribution into whole tissues and cell preparations, without the restrictions required when radioactive isotopes (i.e., ^65^Zn^2+^) would be used. The specificity of these methods for individual metal species not only permits quantification of rates of entry into cells and tissues, but also increases the confidence in designing studies utilizing potentially less specific reporters of Zn^2+^ movements, such as the fluorescent reporter fluozin-3.

### Connection between intracellular Ca^2+^ and uptake of Zn^2+^ across the basolateral membrane of the gastric parietal cell

Based on studies with ^70^Zn^2+^, we were able to design experiments to study conditions of uptake under baseline conditions and during exposure to well-recognized secretory agonists, using utilizing relatively inexpensive and rapidly responsive fluorescence imaging and fluorometric methods. Utilizing these methods, we were able to confirm enhanced uptake of Zn^2+^ across the basolateral membrane during secretory stimulation. In addition, we found that manipulation of [Ca^2+^]_i_ homeostasis leads to alterations in the ability of the parietal cell to take up and preserve [Zn^2+^]_i_. Thus, intracellular accumulation of Zn^2+^ is nearly abolished when intracellular stores of Ca^2+^ are depleted by exposure to thapsigargin and Ca^2+^-depleted Ringer's. Following exposure to thapsigargin, uptake of Zn^2+^ was observed—albeit at a reduced level—if Ca^2+^ is present in the extracellular solution. This uptake was arrested when the chelator DTPA (10 µM) was added. These observations indicate that the increases in [Zn^2+^]_i_ are indeed due to influx from extracellular sources and not release from intracellular stores. They argue that, under baseline conditions, uptake of Zn^2+^ across the basolateral membrane depends on adequate stores of intracellular Ca^2+^. With stimulation by powerful agonists such as forskolin and carbachol, demand for extracellular Zn^2+^ increases and depends on influx of extracellular Ca^2+^. In addition, these studies illustrate physiologically relevant approaches for using both real-time imaging of individual glands, which is time-intensive, and 96-well platforms, which would be amenable to rapid through-put approaches for small molecule screens.

### Additional considerations: technical

The fluorescent reporter utilized here was fluozin-3, which has been utilized in its free acid form for monitoring extracellular secretion of Zn^2+^ from insulin-secreting beta cells [30]. When conjugated to an acetoxymethyl ester group, it has proven useful for monitoring intracellular concentrations of free Zn^2+^ [Zn^2+^] in different cell types [29], [49], including epithelial cells of the gastrointestinal tract [16], [18]. Importantly, this reporter is unresponsive to Ca^2+^ under a number of experimentally relevant conditions [18], [49], [50]. However, it is possible for release or sequestration of other divalent cations to influence responses of the reporter [51]. It is for this reason that caution must be used in applying relationships that have been used to calculate free concentration of Zn^2+^ from the reported *K_d_* and from maximum and minimum fluorescence responses, as was originally proposed for Ca^2+^ reporters [52]. Thus, we have refrained from providing direct estimates of [Zn^2+^]_i_, based on fluozin-3 measurements.

### Additional considerations: connection between intracellular Ca^2+^ and movements of Zn^2+^ across the basolateral membrane of the gastric parietal cell

In the current set of studies, we find that Ca^2+^ facilitates optimal uptake of Zn^2+^ across the cell membrane, implying that it is either a counter-ion in exchange or it is acting as a regulatory second-messenger. Membrane proteins that facilitate Zn^2+^ transport constitute the SLC30A (ZnT) and SLC39A (Zip) gene families [53], [54]. To date, fourteen proteins that facilitate import of Zn^2+^ to the cytoplasm (either from extracellular sources or possibly intracellular compartments) have been identified in mammals [53], [54]. Current information on the mechanisms of Zn^2+^ transport does not implicate Ca^2+^ in the former role and emerging information for ZIP, ZnT and related transporters in yeast suggest that counter exchanging ions are likely to be protons [55]. Based on the well-recognized role of Ca^2+^ as a second messenger in responses to secretagogues [32], [38], [40], [56], [57], [58], [59], our findings offer the novel conclusion that Ca^2+^ is a second messenger that can match the basolateral demand for Zn^2+^ with the secretory response to physiologic stimulation. It seems likely that similar connections will be found in other secretory cells and tissues—neural, endocrine and exocrine.

### Additional considerations: pathophysiological implications of Ca^2+^-regulated Zn^2+^ uptake

Compared to levels of free Ca^2+^, the amount of free Zn^2+^ within the cytoplasm is even more tightly controlled. Our studies have suggested that, in different epithelial cells, the baseline concentration of [Zn^2+^]_i_ is in the nanomolar range [16], [18]. This consideration alone suggests tight regulation of the activities of transporters that modulate demand for Zn^2+^ and its disposal from the cytoplasm. Observations in other cell types such as neurons suggest that exposure to elevated levels of Zn^2+^ can be cytotoxic, within minutes [60], [61]. Preliminary studies in epithelial cells of the gastrointestinal tract confirm that oxidant stress induces increases in [Zn^2+^]_i_ [16] that can influence pathways of cell death and the balance between necrosis and apoptosis [19], [62]. Dysregulation of Ca^2+^ homeostasis is also a consequence of such oxidant-related stress [16], [39]. The findings of the current study suggest that oxidative dysregulation of intracellular Zn^2+^ could be amplified by dysregulation of intracellular Ca^2+^ homeostasis. Further studies will help to clarify this potential relationship, not only in gastric mucosa but in other secretory cell types or tissues.

## References

[1] Jou MYHA, Philipps AF, Kelleher SL, Lönnerdal B (2009). Tissue-specific alterations in zinc transporter expression in intestine and liver reflect a threshold for homeostatic compensation during dietary zinc deficiency in weanling rats.. J Nutr.

[2] Lönnerdal B, Kelleher SL (2009). Micronutrient transfer: infant absorption.. Advances in Experimental Medicine and Biology.

[3] Jou MY, Philipps AF, Kelleher SL, Lönnerdal B (2010). Effects of Zinc Exposure on Zinc Transporter Expression in Human Intestinal Cells of Varying Maturity.. Journal of Pediatric Gastroenterology and Nutrition.

[4] Sempértegui F, Díaz M, Mejía R, Rodríguez-Mora OG, Rentería E (2007). Low concentrations of zinc in gastric mucosa are associated with increased severity of Helicobacter pylori-induced inflammation.. Helicobacter.

[5] Dovhanj J, Kljaic K, Vcev A, Ilakovac V (2010). Helicobacter pylori and trace elements.. Clinical Laboratory.

[6] Carter JW, Lancaster H, Hardman WE, Cameron IL (1997). Zinc deprivation promotes progression of 1,2-dimethylhydrazine-induced colon tumors but reduces malignant invasion in mice.. Nutrition and Cancer.

[7] Fong LY, Mancini R, Nakagawa H, Rustgi AK, Huebner K (2003). Combined cyclin D1 overexpression and zinc deficiency disrupts cell cycle and accelerates mouse forestomach carcinogenesis.. Cancer Research.

[8] Fong LY, Ishii H, Nguyen VT, Vecchione A, Farber JL (2003). p53 deficiency accelerates induction and progression of esophageal and forestomach tumors in zinc-deficient mice.. Cancer Research.

[9] Hoque KM, Binder HJ (2006). Zinc in the treatment of acute diarrhea: current status and assessment.. Gastroenterology.

[10] Patel A, Mamtani M, Dibley MJ, Badhoniya N, Kulkarni H (2010). Therapeutic value of zinc supplementation in acute and persistent diarrhea: a systematic review.. PLoS One.

[11] Frommer DJ (1975). The healing of gastric ulcers by zinc sulphate.. Medical Journal of Australia.

[12] Jiménez E, Bosch F, Galmés JL, Baños JE (1992). Meta-analysis of efficacy of zinc acexamate in peptic ulcer.. Digestion.

[13] Sturniolo GC, Fries W, Mazzon E, Di Leo V, Barollo M (2002). Effect of zinc supplementation on intestinal permeability in experimental colitis.. Journal of Laboratory and Clinical Medicine.

[14] Scrimgeour AG, Condlin ML (2009). Zinc and micronutrient combinations to combat gastrointestinal inflammation.. Current Opinion in Clinical Nutrition and Metabolic Care.

[15] Gerbino A, Hofer AM, McKay B, Lau BW, Soybel DI (2004). Divalent cations regulate acidity within the lumen and tubulovesicle compartment of gastric parietal cells.. Gastroenterology.

[16] Cima RR, Dubach JM, Wieland AM, Walsh BM, Soybel DI (2006). Intracellular Ca^2+^ and Zn^2+^ signals during monochloramine-induced oxidative stress in isolated rat colon crypts.. American Journal of Physiology: Gastrointestinal and Liver Physiology.

[17] Yu YY, Kirschke CP, Huang L (2007). Immunohistochemical analysis of ZnT1, 4, 5, 6, and 7 in the mouse gastrointestinal tract.. Journal of Histochemistry and Cytochemistry.

[18] Naik HB, Beshire M, Walsh BM, Liu J, Soybel DI (2009). Secretory State regulates Zn^2+^ Transport in the Gastric Parietal Cell of the Rabbit.. American Journal of Physiology: Cell and Molecular Physiology.

[19] Kohler JE, Dubach JM, Naik HB, Tai K, Blass AL (2010). Monochloramine-induced toxicity and dysregulation of intracellular Zn^2+^ in parietal cells of rabbit gastric glands.. American Journal of Physiology: Gastrointestinal and Liver Physiology.

[20] Hidalgo M, Eckhardt SG (2001). Development of matrix metalloproteinase inhibitors in cancer therapy.. Journal of the National Cancer Institute.

[21] Azriel-Tamir H, Sharir H, Schwartz B, Hershfinkel M (2004). Extracellular zinc triggers ERK-dependent activation of Na^+^/H^+^ exchange in colonocytes mediated by the zinc-sensing receptor.. Journal of Biological Chemistry.

[22] Kirchhoff P, Socrates T, Sidani S, Duffy A, Breidthardt T (2010). Zinc Salts Provide a Novel, Prolonged and Rapid Inhibition of Gastric Acid Secretion American Journal of Gastroenterology Aug 24 [Epub ahead of print]..

[23] Joseph RM, Varela V, Kanji VK, Subramony C, Mihas AA (1999). Protective effects of zinc in indomethacin-induced gastric mucosal injury: evidence for a dual mechanism involving lipid peroxidation and nitric oxide.. Alimentary Pharmacology and Therapeutics.

[24] Mahmood A, FitzGerald AJ, Marchbank T, Ntatsaki E, Murray D (2007). Zinc carnosine, a health food supplement that stabilizes small bowel integrity and stimulates gut repair processes.. Gut.

[25] Berglindh T, Helander HF, Obrink KJ (1976). Effects of secretagogues on oxygen consumption, aminopyrine accumulation and morphology in isolated gastric glands.. Acta Physiologica Scandinavia.

[26] Hunter DB, Bertsch PM (2001). Applications of Synchrotron-Based X-ray Microprobes.. Chemical Reviews.

[27] Marcus MA, MacDowell AA, Celestre R, Manceau A, Miller T (2004). Beamline 10.3.2 at ALS: a hard X-ray microprobe for environmental and materials sciences.. Journal of Synchrotron Radiation.

[28] Amarasiriwardena CJ, Krushevska A, Foner H, Argentine MD, Barnes RM (1992). Inductively Coupled Plasma Mass Spectrometric Determination of 70Zn to 68Zn Isotope Ratio in Biological Samples from Pre-term Human Babies.. Journal of Analytical Atomic Spectrometry.

[29] Devinney MJ, Reynolds IJ, Dineley KE (2005). Simultaneous detection of intracellular free calcium and zinc using fura-2FF and fluozin-3.. Cell Calcium.

[30] Gee KR, Zhou ZL, Qian WJ, Kennedy R (2002). Detection and imaging of zinc secretion from pancreatic beta cells using a new fluorescent zinc indicator.. Journal of the American Chemical Society.

[31] Helander HF, Hirschowitz BI (1974). Quantitative ultrastructural studies on inhibited and on partly stimulated gastric parietal cells.. Gastroenterology.

[32] Berglindh T, Obrink KJ (1976). A method for preparing isolated glands from the rabbit gastric mucosa.. Acta Physiologica Scandinavia.

[33] Janghorbani M, Ting BT, Istfan NW, Young VR (1981). Measurement of ^68^Zn and ^70^Zn in human blood in reference to the study of zinc metabolism.. American Journal of Clinical Nutrition.

[34] Maret W (2001). Crosstalk of the group IIa and IIb metals calcium and zinc in cellular signaling.. Proceedings of the National Academy of Sciences, USA.

[35] Magneson GR, Puvathingal JM, Ray WJJ (1987). The concentrations of free Mg^2+^ and free Zn^2+^ in equine blood plasma.. Journal of Biological Chemistry.

[36] Mathew J, Kohler JE, Blass AL, Kelly E, Soybel DI (2010). A Novel Assay for Plasma Free [Zn^2+^] in a Rat Model of Hemorrhagic Shock Journal of Surgical Research.

[37] Arslan P, Di Virgilio F, Beltrame M, Tsien RY, Pozzan T (1985). Cytosolic Ca^2+^ homeostasis in Ehrlich and Yoshida carcinomas. A new, membrane-permeant chelator of heavy metals reveals that these ascites tumor cell lines have normal cytosolic free Ca^2+^.. Journal of Biological Chemistry.

[38] Negulescu PA, Machen TE (1993). Ca^2+^ transport by plasma membrane and intracellular stores of gastric cells.. American Journal of Physiology: Cell and Molecular Physiology.

[39] Walsh BM, Naik HB, Dubach JM, Beshire M, Wieland AM (2007). Thiol-oxidant monochloramine mobilizes intracellular Ca^2+^ in parietal cells of rabbit gastric glands.. American Journal of Physiology: Cell and Molecular Physiology.

[40] Hofer AM, Machen TE (1993). Technique for in situ measurement of calcium in intracellular inositol 1,4,5-trisphosphate-sensitive stores using the fluorescent indicator mag-fura-2.. Proceedings of the National Academy of Sciences, USA.

[41] Chew CS (1983). Forskolin stimulation of acid and pepsinogen secretion in isolated gastric glands.. American Journal of Physiology: Cell and Molecular Physiology.

[42] Hersey SJ, Steiner L (1985). Acid formation by permeable gastric glands: enhancement by prestimulation.. American Journal of Physiology: Gastrointestinal and Liver Physiology.

[43] Wagner D, Maser J, Lai B, Cai Z, Barry CE (2005). Elemental analysis of Mycobacterium avium-, Mycobacterium tuberculosis-, and Mycobacterium smegmatis-containing phagosomes indicates pathogen-induced microenvironments within the host cell's endosomal system.. J Immunol.

[44] Yang L, McRae R, Henary MM, Patel R, Lai B (2005). Imaging of the intracellular topography of copper with a fluorescent sensor and by synchrotron x-ray fluorescence microscopy.. Proc Natl Acad Sci U S A.

[45] Finney L, Mandava S, Ursos L, Zhang W, Rodi D (2007). X-ray fluorescence microscopy reveals large-scale relocalization and extracellular translocation of cellular copper during angiogenesis.. Proc Natl Acad Sci U S A.

[46] McCormick N, Velasquez V, Finney L, Vogt S, Kelleher SL (2010). X-ray fluorescence microscopy reveals accumulation and secretion of discrete intracellular zinc pools in the lactating mouse mammary gland.. PLoS One.

[47] Ito S, Code CF (1967). Anatomic structure of the gastric mucosa.. Alimentary Canal: Secretion.

[48] deFoneska A, Kaunitz JD (2010). Gastroduodenal mucosal defense.. Curr Opin Gastroenterol.

[49] Martin JL, Stork CJ, Li YV (2006). Determining zinc with commonly used calcium and zinc fluorescent indicators, a question on calcium signals.. Cell Calcium.

[50] Jia Y, Jeng JM, Sensi SL, Weiss JH (2002). Zn^2+^ currents are mediated by calcium-permeable AMPA/kainate channels in cultured murine hippocampal neurones.. Journal of Physiology.

[51] Haugland RP (2003). Molecular Probes Product Information and Catalogue.

[52] Grynkiewicz G, Poenie M, Tsien RY (1985). A new generation of Ca^2+^ indicators with greatly improved fluorescence properties.. Journal of Biological Chemistry.

[53] Guerinot ML (2000). The ZIP family of metal transporters.. Biochimica et Biophysica Acta.

[54] Lichten LA, Cousins RJ (2009). Mammalian zinc transporters: nutritional and physiologic regulation.. Annual Review of Nutrition.

[55] Ohana E, Hoch E, Keasar C, Kambe T, Yifrach O (2009). Identification of the Zn^2+^ binding site and mode of operation of a mammalian Zn^2+^ transporter.. Journal of Biological Chemistry.

[56] Caroppo R, Gerbino A, Debellis L, Kifor O, Soybel DI (2001). Asymmetrical, agonist-induced fluctuations in local extracellular [Ca^2+^] in intact polarized epithelia.. EMBO Journal.

[57] Geibel JP, Wagner CA, Caroppo R, Qureshi I, Gloeckner J (2001). The stomach divalent ion-sensing receptor SCAR is a modulator of gastric acid secretion.. Journal of Biological Chemistry.

[58] Muallem S, Sachs G (1985). Ca^2+^ metabolism during cholinergic stimulation of acid secretion.. American Journal of Physiology: Gastrointestinal and Liver Physiology.

[59] Perez-Zoghbi JF, Mayora A, Ruiz MC, Michelangeli F (2008). Heterogeneity of acid secretion induced by carbachol and histamine along the gastric gland axis and its relationship to [Ca^2+^]_i_.. American Journal of Physiology: Gastrointestinal and Liver Physiology.

[60] Yokoyama M, Koh JY, Choi DW (1986). Brief exposure to zinc is toxic to cortical neurons.. Neuroscience Letters.

[61] Koh JY, Choi DW (1994). Zinc toxicity on cultured cortical neurons: involvement of N-methyl-D-aspartate receptors.. Neuroscience.

[62] Kohler JE, Mathew J, Tai K, Blass AL, Kelly E (2009). Monochloramine impairs caspase-3 through thiol oxidation and Zn^2+^ release.. Journal of Surgical Research.

